# Supplementary material for: Association between seasonal respiratory virus activity and invasive pneumococcal disease in central Ontario, Canada
Source: BMC Infect Dis. 2026 Jan 24;26:375. doi: 10.1186/s12879-025-12419-8 (PMC12911223; doi:10.1186/s12879-025-12419-8)
Supplement: Supplementary file 1 — Supplementary Material 1 [file 12879_2025_12419_MOESM1_ESM.docx]

**SUPPLEMENTARY APPENDIX**

| M | Tu | W | Th | F | Sa | Su | M | Tu | W | Th | F | Sa | Su | M | Tu | W | Th | F | Sa | Su |
| --- | --- | --- | --- | --- | --- | --- | --- | --- | --- | --- | --- | --- | --- | --- | --- | --- | --- | --- | --- | --- |

Control

Control

Case

| M | Tu | W | Th | F | Sa | Su | M | Tu | W | Th | F | Sa | Su | M | Tu | W | Th | F | Sa | Su |
| --- | --- | --- | --- | --- | --- | --- | --- | --- | --- | --- | --- | --- | --- | --- | --- | --- | --- | --- | --- | --- |

Control

Case

Control

| M | Tu | W | Th | F | Sa | Su | M | Tu | W | Th | F | Sa | Su | M | Tu | W | Th | F | Sa | Su |
| --- | --- | --- | --- | --- | --- | --- | --- | --- | --- | --- | --- | --- | --- | --- | --- | --- | --- | --- | --- | --- |

Control

Control

Case

**Supplementary Figure 1.** Diagram of control selection for our matched 2:1 case-crossover study, adapted from Berry et al., 2020 (7). Controls were matched with cases by day of the week and could occur up to two weeks before or after a case.

**
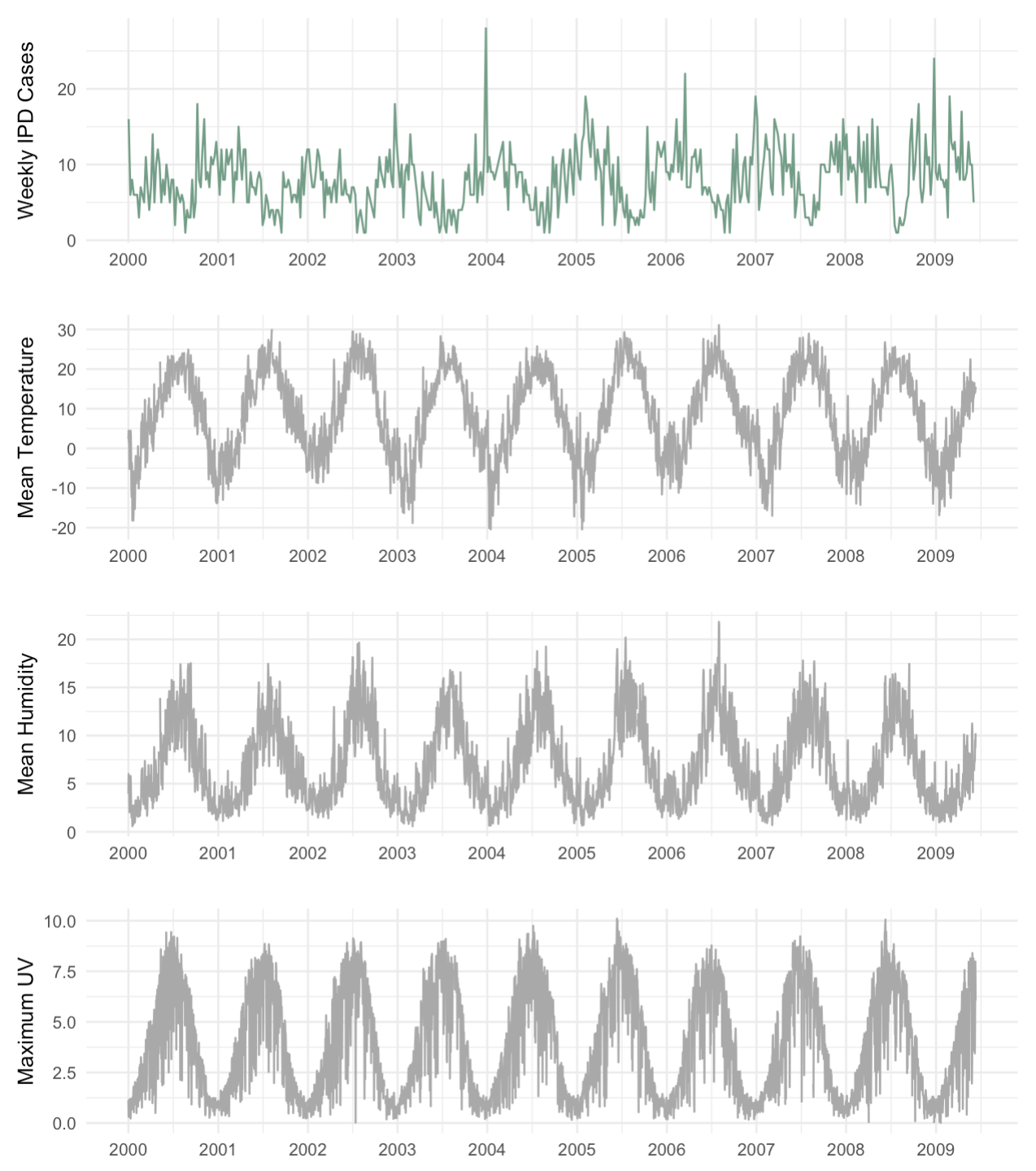
**

*Notes:* The x-axis labels align with January of each year. Each graph has a different y-axis scale.

**Supplementary Figure 2.** Daily mean temperature ($℃$), mean absolute humidity ($g/m^{3}$), and maximum UV index in Central Ontario, Canada

**Supplementary Table 1**. Odds ratios (OR) and 95% confidence intervals (95% CI) of the relationship between weekly respiratory virus activity (per 100 viral isolations) and invasive pneumococcal disease (IPD). A hazard period of one to seven days between respiratory virus activity and IPD was used. Absolute humidity, UV index, and temperature were included as covariates.

| **Exposure** | **OR (95% CI)** | ***P*-value** |
| --- | --- | --- |
| RSV | 0.75 (0.58, 0.99) | *p <* 0.05 |
| Influenza A | 1.07 (0.95, 1.21) | *p* = 0.26 |
| Influenza B | 1.65 (0.84, 3.22) | *p* = 0.15 |
| RSV $*$ Influenza A | 1.11 (0.96, 1.29) | *p* = 0.16 |
| RSV $*$ Influenza B | 1.24 (0.81, 1.89) | *p* = 0.32 |
| Influenza A $*$ Influenza B | 0.71 (0.54, 0.92) | *p <* 0.05 |
| Mean Temperature ($℃$) | 1.01 (0.99, 1.04) | *p* = 0.24 |
| Mean Absolute Humidity ($g/m^{3}$) | 0.97 (0.92, 1.03) | *p* = 0.18 |
| Maximum UV Index | 0.95 (0.89, 1.02) | *p* = 0.31 |

**Supplementary Table 2**. Odds ratios (OR) and 95% confidence intervals (95% CI) of the relationship between weekly respiratory virus activity (per 100 viral isolations) and invasive pneumococcal disease (IPD). A hazard period of eight to ten days between respiratory virus activity and IPD was used. Absolute humidity, UV index, and temperature were included as covariates.

| **Exposure** | **OR (95% CI)** | ***P*-value** |
| --- | --- | --- |
| RSV | 1.03 (0.81, 1.32) | *p* = 0.79 |
| Influenza A | 1.05 (0.94, 1.18) | *p* = 0.37 |
| Influenza B | 1.43 (0.75, 2.72) | *p* = 0.28 |
| RSV $*$ Influenza A | 1.01 (0.88, 1.17) | *p* = 0.84 |
| RSV $*$ Influenza B | 0.79 (0.53, 1.17) | *p* = 0.24 |
| Influenza A $*$ Influenza B | 1.03 (0.82, 1.30) | *p* = 0.81 |
| Mean Temperature ($℃$) | 0.99 (0.97, 1.00) | *p* = 0.17 |
| Mean Absolute Humidity ($g/m^{3}$) | 1.01 (0.97, 1.05) | *p* = 0.58 |
| Maximum UV Index | 1.10 (1.05, 1.16) | *p <* 0.001 |

**Supplementary Table 3**. Odds ratios (OR) and 95% confidence intervals (95% CI) of the relationship between weekly respiratory virus activity (per 100 viral isolations) and invasive pneumococcal disease (IPD). A hazard period of one to fourteen days between respiratory virus activity and IPD was used. Absolute humidity, UV index, and temperature were included as covariates.

| **Exposure** | **OR (95% CI)** | ***P*-value** |
| --- | --- | --- |
| RSV | 0.90 (0.67, 2.92) | *p* = 0.51 |
| Influenza A | 1.17 (1.01, 1.36) | *p* < 0.05 |
| Influenza B | 1.35 (0.62, 2.92) | *p* = 0.45 |
| RSV $*$ Influenza A | 1.00 (0.83, 1.20) | *p* = 0.99 |
| RSV $*$ Influenza B | 0.98 (0.61, 1.58) | *p* = 0.94 |
| Influenza A $*$ Influenza B | 0.93 (0.68, 1.27) | *p* = 0.66 |
| Mean Temperature ($℃$) | 0.98 (0.95, 1.02) | *p* = 0.36 |
| Mean Absolute Humidity ($g/m^{3}$) | 1.01 (0.93, 1.09) | *p* = 0.87 |
| Maximum UV Index | 1.10 (0.99, 1.22) | *p =* 0.08 |

**Supplementary Table 4**. Odds ratios (OR) and 95% confidence intervals (95% CI) of the relationship between weekly respiratory virus activity (per 100 viral isolations) and invasive pneumococcal disease (IPD). A hazard period of one to twenty-one days between respiratory virus activity and IPD was used. Absolute humidity, UV index, and temperature were included as covariates.

| **Exposure** | **OR (95% CI)** | ***P*-value** |
| --- | --- | --- |
| RSV | 0.88 (0.63, 1.22) | *p* = 0.44 |
| Influenza A | 1.20 (1.02, 1.41) | *p <* 0.05 |
| Influenza B | 1.38 (0.58, 3.26) | *p* = 0.47 |
| RSV $*$ Influenza A | 0.98 (0.80, 1.21) | *p* = 0.87 |
| RSV $*$ Influenza B | 0.78 (0.46, 1.32) | *p* = 0.35 |
| Influenza A $*$ Influenza B | 1.04 (0.73, 1.49) | *p* = 0.81 |
| Mean Temperature ($℃$) | 0.97 (0.92, 1.01) | *p* = 0.13 |
| Mean Absolute Humidity ($g/m^{3}$) | 1.02 (0.92, 1.13) | *p* = 0.71 |
| Maximum UV Index | 1.11 (0.97, 1.27) | *p* = 0.13 |

**Supplementary Table 5**. Odds ratios (OR) and 95% confidence intervals (95% CI) of the relationship between weekly respiratory virus activity (per 100 viral isolations) and invasive pneumococcal disease (IPD). A hazard period of one to twenty-eight days between respiratory virus activity and IPD was used. Absolute humidity, UV index, and temperature were included as covariates.

| **Exposure** | **OR (95% CI)** | ***P*-value** |
| --- | --- | --- |
| RSV | 0.92 (0.65, 1.30) | *p* = 0.62 |
| Influenza A | 1.19 (0.99, 1.43) | *p* = 0.07 |
| Influenza B | 1.29 (0.50, 3.30) | *p* = 0.60 |
| RSV $*$ Influenza A | 0.99 (0.79, 1.24) | *p* = 0.91 |
| RSV $*$ Influenza B | 0.78 (0.44, 1.39) | *p* = 0.40 |
| Influenza A $*$ Influenza B | 1.05 (0.70, 1.58) | *p* = 0.81 |
| Mean Temperature ($℃$) | 0.97 (0.92, 1.03) | *p* = 0.35 |
| Mean Absolute Humidity ($g/m^{3}$) | 0.99 (0.87, 1.13) | *p* = 0.87 |
| Maximum UV Index | 1.11 (0.94, 1.30) | *p* = 0.22 |

**Supplementary Table 6**. Odds ratios (OR) and 95% confidence intervals (95% CI) of the relationship between weekly respiratory virus activity (per 100 viral isolates) and invasive pneumococcal disease (IPD) from November 1^st^ to April 30^th^. A hazard period of one to three days between respiratory virus activity and IPD was used.

|  | **Univariable models** | | **Single viral exposure, adjusted for environmental covariates*^a^*** | | **Multivariable model with all viral exposures, adjusted for environmental covariates*^a^*** | |
| --- | --- | --- | --- | --- | --- | --- |
| **Viral exposure** | **OR**  **(95% CI)** | ***P*-value** | **OR**  **(95% CI)** | ***P*-value** | **OR**  **(95% CI)** | ***P*-value** |
| RSV | 1.02  (0.83, 1.25) | *p* = 0.86 | 1.00  (1.00, 1.00) | *p* = 0.88 | 0.97  (0.79, 1.19) | *p* = 0.75 |
| Influenza A | 1.10  (1.00, 1.21) | *p* < 0.05 | 1.10  (1.00, 1.20) | p = 0.06 | 1.08  (0.98, 1.19) | *p* = 0.11 |
| Influenza B | 1.42  (1.03, 1.95) | *p* < 0.03 | 1.45  (1.05, 2.00) | *p* < 0.02 | 1.40  (1.01, 1.94) | *p* < 0.05 |

*^a^* Environmental covariates include mean temperature, mean absolute humidity, and maximum UV index

**Supplementary Table 7**. Odds ratios (OR) and 95% confidence intervals (95% CI) of the relationship between weekly respiratory virus activity (per 100 viral isolates) and invasive pneumococcal disease (IPD) from November 1^st^ to April 30^th^. A hazard period of one to three days between respiratory virus activity and IPD was used. Absolute humidity, UV index, and temperature were included as covariates.

| **Exposure** | **OR (95% CI)** | ***P*-value** |
| --- | --- | --- |
| RSV | 0.88 (0.68, 1.14) | *p* = 0.35 |
| Influenza A | 1.02 (0.89, 1.17) | *p* = 0.75 |
| Influenza B | 2.84 (1.40, 5.74) | *p* < 0.01 |
| RSV $*$ Influenza A | 1.15 (0.99, 1.33) | p = 0.07 |
| RSV $*$ Influenza B | 0.89 (0.58, 1.35) | *p* = 0.58 |
| Influenza A $*$ Influenza B | 0.70 (0.55, 0.90) | *p* < 0.01 |
| Mean Temperature ($℃$) | 1.01 (0.98, 1.03) | *p* = 0.70 |
| Mean Absolute Humidity ($g/m^{3}$) | 0.98 (0.91, 1.07) | *p* = 0.71 |
| Maximum UV Index | 0.96 (0.88, 1.34) | *p* = 0.29 |
